# Clinical parameters related to metamorphopsia outcome in patients with resolved central serous chorioretinopathy using M-CHARTS: retrospective cohort study

**DOI:** 10.1186/s12886-015-0170-4

**Published:** 2015-12-18

**Authors:** Seokhyun Bae, Kiwon Jin, Hakyoung Kim, So Hyun Bae

**Affiliations:** Department of Ophthalmology, Kangnam Sacred Heart Hospital, Hallym University, Singil-ro 1, Yeongdeungpo-gu, 150-950 Seoul, Korea

**Keywords:** Central serous chorioretinopathy, M-CHARTS, Metamorphopsia

## Abstract

**Background:**

The purpose of this study was to determine the clinical parameters related to metamorphopsia outcome in patients with resolved central serous chorioretinopathy (CSCR).

**Methods:**

The charts of 36 eyes with resolved CSCR were retrospectively reviewed. We measured metamorphopsia using M-CHARTS after resolution of serous retinal detachment. We analyzed the relationship between metamorphopsia outcome and clinical parameters including age, visual acuity, chronicity of CSCR, symptom duration and several spectral-domain optical coherence tomography findings using univariate and multivariate forward logistic regression analyses.

**Results:**

The M-CHARTS detected metamorphopsia in 19 eyes (52.8 %). In the univariate analysis, the eyes with metamorphopsia were, relative to those without metamorphopsia, significantly associated with a greater proportion of chronic-recurrent CSCR, initial and final irregularities of retinal pigment epithelium, longer symptom duration, thinner final thickness of central fovea and outer nuclear layer, and final disruption of external limiting membrane (ELM), photoreceptor inner and outer segment junction and cone outer segment tip line (*P* = 0.003, 0.037, 0.019, 0.003, 0.013, 0.015, <0.001, 0. 012 and 0.002, respectively). However, in the multivariate analysis, chronic-recurrent CSCR (OR 22.5, *P* = 0.019) and final disrupted ELM (OR 82.6, *P* = 0.004) were the independent clinical parameters related to poor metamorphopsia outcome.

**Conclusions:**

Residual metamorphopsia was detected using M-CHARTS in about half of patients (52.8 %) with resolved CSCR. Chronic-recurrent CSCR and final disrupted ELM were the independent clinical parameters related to poor metamorphopsia outcome in patients with resolved CSCR.

## Background

Central serous chorioretinopathy (CSCR) is characterized by serous detachment of the neurosensory retina in the macula which is associated with leakage at the level of the retinal pigment epithelium (RPE). Generally, CSCR is a benign and self-limiting disease with spontaneous resolution of serous retinal detachment (SRD) and recovery of good vision. However, visual performance in patients with CSCR can be compromised even after retinal reattachment. The symptoms include loss of visual acuity, metamorphopsia, micropsia, dyschromatopsia, scotoma, and reduced contrast sensitivity. Many studies have reported clinical features related to visual outcome in patients with CSCR; they include symptom duration [1], baseline visual acuity [1], outer nuclear layer (ONL) thickness [2], as well as the integrities of the photoreceptor inner and outer segment (IS/OS) junction [2–4] and cone outer segment tip (COST) line [4].

Metamorphopsia is a major visual symptom in patients with CSCR as residual metamorphopsia often leads to poor visual quality even after recovery of good visual acuity. Recently, several tools have been developed for quantification of metamorphopsia severity including M-CHARTS (Inami Co., Tokyo, Japan) [5], preferential hyperacuity perimeter (PHP) [6], and others. Nonetheless, few studies have investigated the clinical parameters related to metamorphopsia severity in patients with CSCR [7, 8]. Bae and Chae reported an M-CHARTS-quantified correlation of high pigment epithelial detachment (PED) incidence with metamorphopsia in patients with active CSCR; however they did not analyze the clinical factors related to poor metamorphopsia outcome after the resolution of SRD [7]. Fujita et al., after half-dose verteporfin photodynamic therapy (PDT) in patients with chronic CSCR, found no significant correlation between metamorphopsia severity and the integrities of IS/OS junction and COST line [8]. However, the clinical parameters related to metamorphopsia outcome after resolution of SRD in patients with CSCR still have not been firmly established. The purpose of this study was to determine the clinical parameters related to metamorphopsia outcome after resolution of SRD in patients with CSCR.

## Methods

This is a retrospective cohort study. This study was approved by the Institutional Review Board of Kangnam Sacred Heart Hospital and adhered to the tenets of the Declaration of Helsinki. We retrospectively reviewed the medical records of patients with resolved CSCR from August 2014 to May 2015. Each patient had a documented episode of CSCR diagnosed by both fluorescein angiography (FA) and spectral-domain optical coherence tomography (SD OCT). We excluded the patients with other retinal diseases including age-related macular degeneration, polypoidal choroidal vasculopathy (PCV), idiopathic choroidal neovascularization (CNV), epiretinal membrane (ERM), macular hole, vitreomacular traction, and other ocular diseases including proliferative retinopathies, intraocular inflammation, glaucoma, and amblyopia. Also excluded were patients with a history of any systemic corticosteroid use or of ocular surgery except uncomplicated cataract surgery.

The clinical data collected were age, sex, past ocular history, symptom duration, chronicity of CSCR, and treatment types. On the initial visit, all of the patients underwent complete ophthalmic examinations including measurement of best-corrected visual acuity (BCVA), slit-lamp biomicroscopy, fundus examination, fundus photography, FA, and SD OCT (Cirrus HD-OCT; Carl Zeiss Meditec Inc, Dublin, CA). The final clinical data including BCVA, fundus photography, SD-OCT and M-CHARTS at 6–12 months after resolution of SRD were obtained. Acute CSCR was defined as SRD self-resolving within 6 months of symptom onset. Chronic-recurrent CSCR was diagnosed in patients with visual disturbances persisting for more than 6 months or recurrent episodes of CSCR with multifocal or diffuse RPE decompensation on FA. The patients were divided into two groups based on the M-CHARTS-determined presence or absence of metamorphopsia on the final visit.

The BCVA was converted to the logarithm of the minimal angle of resolution (logMAR). Indocyanine green angiography (ICGA) was conducted to exclude PCV and coexisting CNV. The SD-OCT scans were obtained in the 5-line raster mode using a scan length of 6.0 mm centered on the fovea. Based on each horizontal and vertical SD OCT scan on the initial and final visit, the following foveal microstructures were evaluated: PED, RPE irregularities, height of SRD, central foveal thickness (CFT), thickness of ONL, and the integrities of the external limiting membrane (ELM), photoreceptor IS/OS junction, and COST line. RPE irregularity was defined as a condition including a small RPE bump, RPE hypertrophy or atrophy. The height of SRD as well as CFT and ONL thickness were measured separately with the OCT system’s built-in caliper. Specifically, the height of SRD was measured as the distance between the outer border of the sensory retina and the inner border of the RPE at the fovea; the CFT was defined as the distance between the internal limiting membrane (ILM) and the outer border of the RPE at the fovea; the ONL thickness was measured as the distance between the outer border of the ILM and the ELM at the central fovea. Two separate values were obtained in horizontal and vertical scans, respectively, which were averaged for the statistical analyses. The integrities of the ELM, IS/OS junction and COST lines were graded as intact, a focal disruption within 1000 μm from the foveal center and a broad disruption of more than 1000 μm. All of the SD OCT images were reviewed independently by two investigators (HK and SHB) and a consensus was reached on each of the results. If the patients showed persistent visual symptoms or SRD over 3 months, or if they had a history of multiple recurrences, the treatments, including laser photocoagulation and/or half-fluence PDT, were considered at the discretion of the clinicians (HK and SHB). In cases with obvious focal leakage located more than 500 μm from the fovea on FA, the patient was treated with laser photocoagulation. If laser photocoagulation was not feasible, half-fluence PDT was applied to the area of choroidal hyperpermeability shown in the ICGA using a 6 mg/m^2^ dose of verteporfin followed by delivery of a 689-nm laser with a reduced fluence of 25 J/cm^2^.

In the patients with metamorphopsia, a straight line appeared curved or irregular [5]. On M-CHARTS, straight lines are replaced with dotted lines, and the dot intervals (range: 0.2° - 2.0°) are changed from fine to coarse. With increasing dot interval, the line distortion decreases until the dotted line becomes straight. The visual angle that denoted the dot interval of the line seen as straight was considered as the patient’s M-CHARTS score. When a patient was tested with vertical dotted lines, the result was defined as the vertical M-CHARTS score. After the M-CHARTS were rotated 90°, the horizontal M-CHARTS score also was measured according to horizontal dotted lines. The presence of metamorphopsia subsequently was defined as either a vertical or a horizontal M-CHARTS score equal to or greater than 0.2°. The metamorphopsia severity was evaluated by averaging the vertical and horizontal M-CHARTS scores.

Statistical analyses were performed using SPSS (SPSS version 17.0, SPSS, Inc., Chicago, IL). Comparisons between the two groups formed based on the presence or absence of metamorphopsia were conducted using the Mann–Whitney *U* test for the numerical variables and the Chi-square test or Fisher’s exact test for the categorical variables. The Spearman rank correlation test was used to analyze the relationships between the numerical variables and metamorphopsia severity. This analysis was repeated excluding outliers as a sensitivity analysis. Outliers were defined as averaged M-CHARTS scores smaller than 1.5 times of interquartile range or greater than three times from the rest of the scores. A multivariate forward logistic regression analysis was performed to identify the independent parameters associated with metamorphopsia outcome. The odds ratio (OR) and 95 % confidence interval (CI) were estimated. A P value less than 0.05 was considered statistically significant.

## Results

We diagnosed 41 eyes of 38 patients with resolved CSCR in the study period. However, five cases with resolved CSCR were excluded because they were not followed-up at least 6 months after the complete resolution of SRD. Thus, 36 eyes of 33 patients with resolved CSCR were included in this study. There were 28 men (84.8 %) and 5 women (15.2 %). The mean age was 50.8 ± 8.6 years (range: 36–72 years). Acute CSCR was diagnosed in 14 eyes (38.9 %), and chronic-recurrent CSCR in 22 (61.1 %). The mean logMAR BCVA on the initial visit was 0.2 ± 0.2 (range: 0.0–1.0). Thirteen eyes (36.1 %) showed spontaneous resolution of SRD without any treatment, whereas 23 (63.9 %) underwent treatments to resolve the SRD: single half-fluence PDT in 13 eyes (36.1 %), laser photocoagulation in five (13.9 %), and both PDT and laser photocoagulation in five others (13.9 %). After resolution of SRD, M-CHARTS detected metamorphopsia in 19 eyes (52.8 %), whereas 17 (47.2 %) did not exhibit any abnormalities. The mean vertical and horizontal M-CHARTS scores were 0.4 ± 0.5 and 0.3 ± 0.4, respectively, in the 19 study eyes with residual metamorphopsia on M-CHARTS.

The study eyes were divided into two groups based on the M-CHARTS-detected presence or absence of residual metamorphopsia after resolution of SRD. The comparisons of the demographic and clinical parameters according to the presence or absence of metamorphopsia are summarized in Tables 1, 2 and 3. The ratio of chronic-recurrent CSCR and symptom duration were significantly greater in eyes with metamorphopsia than in those without (*P* = 0.003 and 0.003, respectively) (Table 1). On the initial visit, the incidence of RPE irregularities was significantly greater in the eyes with metamorphopsia than in those without (*P* = 0.037) (Table 2). Among the final clinical parameters, the eyes with metamorphopsia showed significantly thinner CFT and ONL thickness, as well as higher incidences of RPE irregularities and disrupted ELM, IS/OS junction and COST line compared with those without metamorphopsia (*P* = 0. 013, 0. 015, 0. 019, <0.001, 0.012 and 0.002, respectively) (Table 3).

**Table 1:** Demographic parameters according to the M-CHARTS-detected metamorphopsia in patients with resolved central serous chorioretinopathy

	With metamorphopsia (*n* = 19)	Without metamorphopsia (*n* = 17)	*P* value
Age (years)	49.7 ± 8.7	52.1 ± 8.5	0.357
Sex, men : women (n)	14 : 5	16 : 1	0.182
Diagnosis, acute : chronic-recurrent (n)	3 : 16	11 : 6	0.003
Symptom duration (months)	10.5 ± 6.7	4.5 ± 4.0	0.003
Treatment			
Spontaneous resolution (n, %)	6, 31.6 %	7, 41.2 %	0.549
Focal laser photocoagulation (n, %)	7, 36.8 %	3, 17.6 %	0.199
Photodynamic therapy (n, %)	10, 52.6 %	8, 47.1 %	0.738

**Table 2:** Initial clinical parameters according to the M-CHARTS-detected metamorphopsia in patients with resolved central serous chorioretinopathy

	With metamorphopsia (*n* = 19)	Without metamorphopsia (*n* = 17)	*P* value
BCVA (logMAR)	0.3 ± 0.3	0.2 ± 0.2	0.383
Height of subretinal fluid (μm)	190.6 ± 151.7	220.8 ± 153.0	0.447
Pigment epithelial detachment (n, %)	4, 21.1 %	6, 35.3 %	0.341
RPE irregularities (n, %)	11, 57.9 %	4, 23.5 %	0.037
External limiting membrane			0.25
Intact (n, %)	6, 31.6 %	9, 52.9 %	
Focal disruption (n, %)	5, 26.3 %	5, 29.4 %	
Broad disruption (n, %)	8, 42.1 %	3, 17.6 %	
Photoreceptor IS/OS junction			0.342
Intact (n, %)	0, 0 %	0, 0 %	
Focal disruption (n, %)	6, 31.6 %	8, 47.1 %	
Broad disruption (n, %)	13, 68.4 %	9, 52.9 %	
COST line			0.09
Intact (n, %)	0, 0 %	0, 0 %	
Focal disruption (n, %)	3, 15.8 %	7, 41.2 %	
Broad disruption (n, %)	16, 84.2 %	10, 58.8 %	

*BCVA* best-corrected visual acuity, *COST* cone outer segment tip, *IS/OS* inner and outer segment, *logMAR* logarithm of the minimal angle of resolution, *RPE* retinal pigment epithelium

**Table 3:** Final clinical parameters according to the M-CHARTS-detected metamorphopsia in patients with resolved central serous chorioretinopathy

	With metamorphopsia (*n* = 19)	Without metamorphopsia (*n* = 17)	*P* value
BCVA (logMAR)	0.1 ± 0.3	0.1 ± 0.2	0.312
Central foveal thickness (μm)	159.4 ± 37.4	187.5 ± 32.6	0.013
Outer nuclear layer thickness (μm)	74.7 ± 25.0	94.5 ± 18.6	0.015
Pigment epithelial detachment (n, %)	2, 10.5 %	4, 23.5 %	0.391
RPE irregularities (n, %)	13, 68.4 %	5, 29.4 %	0.019
External limiting membrane			<0.001
Intact (n, %)	2, 10.5 %	13, 76.5 %	
Focal disruption (n, %)	6, 31.6 %	2, 11.8 %	
Broad disruption (n, %)	11, 57.9 %	2, 11.8 %	
Photoreceptor IS/OS junction			0.012
Intact (n, %)	0, 0 %	7, 41.2 %	
Focal disruption (n, %)	6, 31.6 %	5, 29.4 %	
Broad disruption (n, %)	13, 68.4 %	5, 29.4 %	
COST line			0.002
Intact (n, %)	0, 0 %	0, 0 %	
Focal disruption (n, %)	2, 10.5 %	10, 58.8 %	
Broad disruption (n, %)	17, 89.5 %	7, 41.2 %	

*BCVA* best-corrected visual acuity, *COST* cone outer segment tip; *IS/OS* inner and outer segment, *logMAR* logarithm of the minimal angle of resolution, *RPE* retinal pigment epithelium

**Fig. 1:**
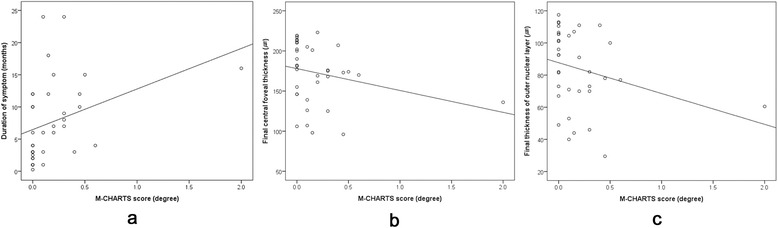
Scattergrams showing correlations between metamorphopsia severity and clinical parameters including symptom duration (**a**), final central foveal thickness (CFT) (**b**) and final thickness of outer nuclear layer (ONL) (**c**) for the 36 total study eyes with resolved central serous chorioretinopathy. The metamorphopsia severity was evaluated by averaging the vertical and horizontal M-CHARTS scores. The M-CHARTS scores showed significant correlation with symptom duration (ρ = 0.517, *P* = 0.001), final CFT (ρ = −0.383, *P* = 0. 021) and final thickness of ONL (ρ = −0.372, *P* = 0.025)

Metamorphopsia severity was significantly correlated with symptom duration, final CFT and final ONL thickness for the 36 total study eyes (ρ = 0.517, *P* = 0.001; ρ = −0.383, *P* = 0.021; ρ = − 0.372, *P* = 0.025; respectively) (Fig. 1), whereas no clinical parameters were significantly correlated with metamorphopsia severity in the 19 eyes with residual metamorphopsia. One outlier was identified with a mean M-CHARTS score of 2.0. The case with extreme outlier showed extensive disruption of ELM, IS/OS junction and COST line finally with a prolonged symptom duration of 16 months. After the removal of identified outlier, the results were similar to those with outlier as follows: metamorphopsia severity was significantly correlated with symptom duration, final CFT and final ONL thickness (ρ = 0.482, *P* = 0.003; ρ = −0.351, *P* = 0.039; ρ = − 0.339, *P* = 0.047; respectively).

In the multivariate analysis, there were two independent clinical parameters associated with poor metamorphopsia outcome: chronic-recurrent CSCR (OR 22.5, 95 % CI 1.7–306.3, *P* = 0.019) and disrupted ELM on the final visit. The eyes with disrupted ELM were associated with a higher risk of poor metamorphopsia outcome relative to those with intact ELM: focal (OR 25.4, 95 % CI 1.6–394.2, *P* = 0.021) or broad disrupted ELM (OR 82.6, 95 % CI 4.2–1630.0, *P* = 0.004).

**Fig. 2:**
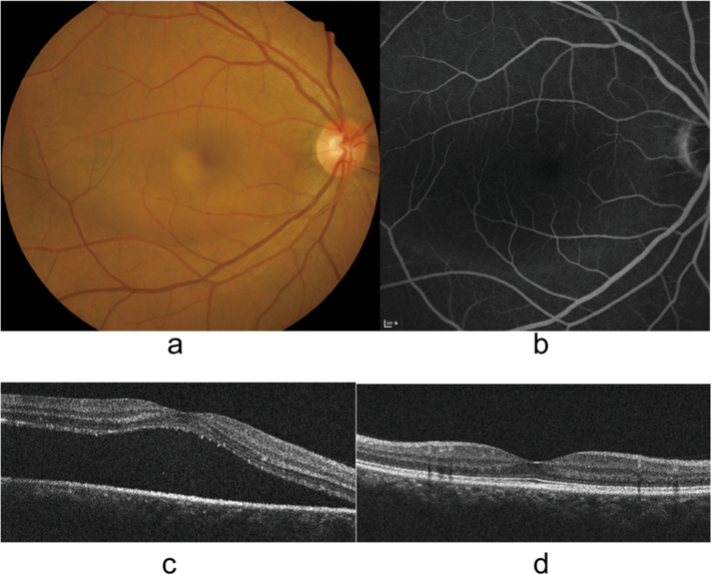
Representative case of a 52-year-old man with acute central serous chorioretinopathy. Baseline fundus photography showed serous retinal detachment (SRD) at the posterior pole (**a**). Baseline fluorescein angiography showed faint leakage at the level of retinal pigment epithelium (**b**). Baseline cross-sectional image of spectral-domain optical coherence tomography (SD OCT) showed the SRD (**c**). The SRD was completely regressed within 2 months of symptom onset without any treatment. At 8 months after complete resolution of SRD, the metamorphopsia was not detected by M-CHARTS. The final SD OCT image showed intact external limiting membrane and photoreceptor inner and outer segment junction (**d**). However, cone outer segment tip line was obscure

**Fig. 3:**
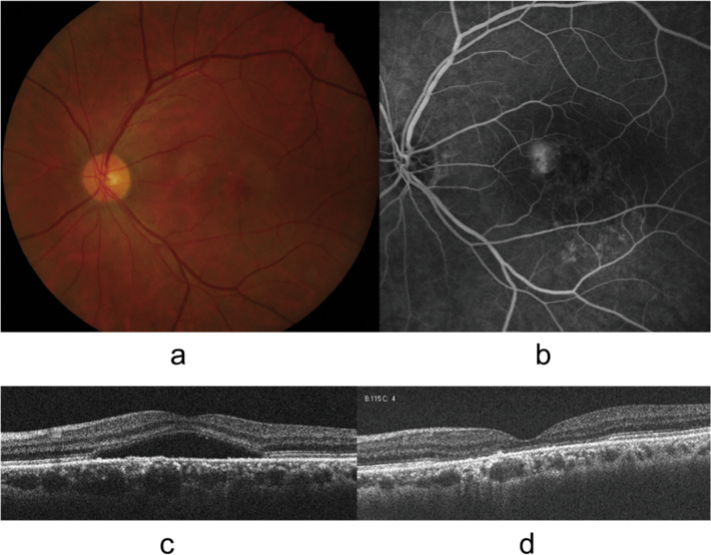
Representative case of a 52-year-old man with chronic central serous chorioretinopathy. Baseline fundus photography showed serous retinal detachment (SRD) at the posterior pole (**a**). Baseline fluorescein angiography showed multiple mottled hyperfluorescent lesions at the macula (**b**). Baseline cross-sectional image of spectral-domain optical coherence tomography (SD OCT) showed the SRD (**c**). The SRD was completely regressed after half-fluence photodynamic therapy. At 6 months after complete resolution of SRD, the metamorphopsia was detected by M -CHARTS with his vertical and horizontal M-CHARTS scores of 0.4 and 0.2, respectively. The final SD OCT image showed broad disruption of external limiting membrane, photoreceptor inner and outer segment junction and cone outer segment tip line (**d**)

Two representative cases are shown in Figs. 2 and 3, respectively.

## Discussion

Metamorphopsia is a common and significant symptom of many retinal diseases. Several investigators have analyzed the prognostic factors affecting metamorphopsia outcomes in several retinal diseases. They reported that the CFT [9–11], ONL thickness [12], thickness of inner nuclear layer (INL) [13, 14] and the integrity of IS/OS junction [10] are related to metamorphopsia severity in patients with ERM. Additionally, Bae and Chae reported that higher incidence of PED is correlated with severe metamorphopsia in patients with active CSCR [7]. However, they could not analyze the clinical factors related to poor metamorphopsia outcome after the resolution of SRD, because all patients did not show sustained metamorphopsia after the regression of subretinal fluid [7]. Still, the clinical parameters underlying metamorphopsia remain controversial.

The present study investigated the clinical parameters associated with metamorphopsia outcome in patients with resolved CSCR. Metamorphopsia severity was quantified using M-CHARTS, and clinical parameters including the foveal microstructures were evaluated on the basis of SD OCT images. Metamorphopsia was detected in about half of the eyes (52.8 %) with resolved CSCR using M-CHARTS, which result is compatible with an incidence up to 67.7 % determined using the Amsler chart [15]. Comparisons between the two groups with and without metamorphopsia showed that metamorphopsia group was correlated with chronic-recurrent CSCR and longer symptom duration as well as several SD-OCT findings as follows: presence of initial and final RPE irregularities, thinner final CFT and ONL thickness, and disrupted ELM, IS/OS junction and COST line on the final visit. Some of these findings are discordant with the previous studies. Fujita et al. found no significant correlation between metamorphopsia severity and the integrities of the IS/OS junction and COST line after half-dose verteporfin PDT in chronic CSCR [8]. Okamoto et al. demonstrated metamorphopsia to be unrelated to the degree of ELM, IS/OS junction or COST disruption in patients with ERM [13, 14]. Even so, the present multivariate analysis showed the disappearance of structural parameters (except final integrity of ELM) as prognostic factors. This result might have been due to structural changes, except that final integrity of the ELM does not primarily affect metamorphopsia outcome but merely results from the clinical course of chronic-recurrent CSCR. Prolonged SRD in patients with chronic-recurrent CSCR could lead to permanent damage to overlying retinal structures, resulting in retinal thinning and disruption of features of the outer retinal layer such as the IS/OS junction, the COST line and the RPE layer, even after resolution of SRD. Ultimately, the key parameters responsible for metamorphopsia outcome, as determined in this study, are chronic-recurrent CSCR and the final integrity of the ELM.

Although several studies have suggested hypotheses on the underlying mechanism of metamorphopsia in several retinal diseases [12–14], in CSCR, it remains unclear. Researchers have speculated that in patients with ERM, the tractional force might damage the inner retinal layer, and primarily the INL [12–14]. A stretched INL, in turn, might cause damage to cellular components such as horizontal, bipolar, amacrine and Müller cells, leading to disturbances in synaptic transmission [12–14]. Whereas, Fujita et al. suggested that in CSCR, metamorphopsia results from disruption to the regular alignment of the photoreceptor layers followed by development of subretinal fluid [8], but their investigation could not produce any corroborating evidence.

The present results showed that final integrity of ELM was the only independent structural parameter impacting on metamorphopsia outcome in resolved CSCR. The ELM represents the border between the outer part of the ONL, composed of photoreceptor cell bodies, and the inner photoreceptor segment. Thus, we speculated that metamorphopsia in resolved CSCR can be attributed to damage to the photoreceptor layer extending toward the photoreceptor cell bodies, not to the outer parts of the photoreceptor layer such as the IS/OS junction or COST line. Furthermore, the ELM offers a junction for Müller cells’ adherence to the base of the outer segment of the photoreceptor layer. The inner half of the foveola is composed of an inverted cone-shaped zone of Müller cells designated as Müller cell cones [16], of which the truncated apex is at the ELM. In addition, Müller cells play a critical role in the control the homeostasis of retinal neurons [17, 18], as well as, by neurotransmitter recycling, the regulation of the synaptic activity in the inner retina [19]. Disruption of the ELM, therefore, might result in dysfunction of Müller cells, leading to disturbances in synaptic transmission, and contributing thereby to the development of metamorphopsia in resolved CSCR.

This study has several limitations among which are the retrospective design and the relatively small sample size. Also, we obtained the final clinical data, including the metamorphopsia measurements, at different time periods after resolution of SRD, even though metamorphopsia and structural parameters could be improved continuously according to the follow-up period. However, in previous studies, the numerical values of M-CHARTS scores and the status of outer retinal layer such as the IS/OS junction, the ELM, and the COST line, were similar at 6–12 months after PDT in patients with chronic CSCR [4, 8]. Thus, the present result might not be significantly influenced by the measurements over different time periods because we evaluated the severity of metamorphopsia at 6–12 months after resolution of SRD. In this study, the structural changes on the SD OCT images were evaluated based only on a few cross-sectional images centered on the fovea. That might be suitable to evaluate the impact on the visual acuity which reflects merely foveal function. However, investigation of a broader retinal area might be necessary in order to evaluate the relationships with metamorphopsia, not just visual acuity. Further studies with larger sample sizes and serial follow-ups will be needed.

## Conclusions

In conclusion, this study detected residual metamorphopsia using M-CHARTS in about half of patients (52.8 %) with resolved CSCR. The independent clinical parameters for poor metamorphopsia outcome were chronic-recurrent CSCR and final disruption of ELM after complete resolution of SRD in patients with CSCR.

### Availability of data and materials

The dataset will not be shared, because there is an additional ongoing study based on the dataset in part.

## Abbreviations

BCVA: best-corrected visual acuity
CFT: central foveal thickness
CI: confidence interval
CNV: choroidal neovascularization
COST: cone outer segment tip
CSCR: central serous chorioretinopathy
ELM: external limiting membrane
ERM: epiretinal membrane
FA: fluorescein angiography
ICGA: indocyanine green angiography
ILM: internal limiting membrane
INL: inner nuclear layer
IS/OS: inner and outer segment
logMAR: Logarithm of the minimal angle of resolution
ONL: outer nuclear layer
OR: odds ratio
PCV: polypoidal choroidal vasculopathy
PDT: photodynamic therapy
PED: pigment epithelial detachment
PHP: preferential hyperacuity perimeter
RPE: retinal pigment epithelium
SD-OCT: spectral-domain optical coherence tomography
SRD: serous retinal detachment
